# *Bacillus velezensis* as a Novel Species for Mosquito Control: Impacts of Exposure on Key Life History Traits of *Culex quinquefasciatus*

**DOI:** 10.3390/insects16040434

**Published:** 2025-04-20

**Authors:** Abdullah A. Alomar

**Affiliations:** Infectious Disease Vector Research Laboratory, Department of Plant Protection, College of Food and Agricultural Sciences, King Saud University, Riyadh 11451, Saudi Arabia; abdalomar@ksu.edu.sa

**Keywords:** *Bacillus velezensis*, mosquito biocontrol, *Culex quinquefasciatus*, larvicidal activity, vector control, sublethal effects, mosquito population suppression

## Abstract

*Bacillus velezensis* is a promising mosquito vector biocontrol agent due to its potent larvicidal properties. This study investigated the lethal and sublethal effects of exposure to crude toxin of *B. velezensis* strain WHk23 on *Culex quinquefasciatus*. Toxicity bioassays showed a median lethal concentration of 28.30 µg/mL for third instar larvae. In addition to direct larval mortality, sublethal exposures resulted in concentration-dependent decreases in survival, fecundity, and fertility. Exposure to different concentrations was found to accelerate larval development, resulting in larger females, yet those females exhibited lower lifespan and diminished reproductive success. These results suggest that exposure to *B. velezensis* not only reduces mosquito populations but also has lasting effects on important life history parameters, ultimately lowering vectorial capacity and disease transmission risk. This study provides valuable insights into the lethal and sublethal consequences of *B. velezensis* exposure. Given its potent larvicidal properties, *B. velezensis* represents a promising biocontrol agent for mosquito vectors.

## Simple Summary

Mosquitoes are considered a major threat to public health as they spread infectious diseases, such as Rift Valley fever, dengue, and West Nile viruses. Traditional chemical insecticides can harm the environment and lead to insecticide resistance, so scientists are looking for safer, more sustainable alternatives. This study examined the use of *Bacillus velezensis*, a naturally occurring bacterium, to control mosquito populations. The bacterium effectively killed *Culex quinquefasciatus* mosquito larvae and reduced their growth, survival, and reproduction following exposure. This means that *B. velezensis* not only directly reduces mosquito populations, but also impairs their ability to survive and reproduce, leading to lower mosquito populations over time. These findings highlight the potential of *B. velezensis* as an environmentally friendly mosquito control method that could help reduce the spread of mosquito-borne diseases while minimizing environmental damage.

## 1. Introduction

The increasing burden of diseases transmitted by mosquitoes, including dengue, Zika, Rift Valley fever, and West Nile viruses, underscores the urgent need for the development of effective and sustainable strategies in disease vector control. In the absence of effective vaccines or treatments for most of these pathogens, vector control programs have heavily depended on the use of chemical insecticides to suppress the population of mosquitoes. However, this dependence on chemicals has raised concerns, as prolonged exposure led to insecticide resistance in the targeted mosquito population and unintended harm to the environments [1]. Therefore, there is growing interest in innovative biological control tools that provide eco-friendly and effective alternatives. These include microbial toxins (e.g., *Bacillus thuringiensis* subsp. *israelensis*), genetically modified mosquitoes, and endosymbiotic bacteria (e.g., *Wolbachia*) that show efficacy in the lowering of mosquito populations and disease transmission [2,3,4,5,6,7]. As research advances, these biological control agents offer a promising path toward integrated and sustainable mosquito control strategies that reduce reliance on chemical insecticides and reduce the risk of insecticide resistance.

*Bacillus velezensis* (*Bv*) is a bacterium that produces a toxin [8]. This Gram-positive spore-forming bacterium is characterized by its ability to produce a variety of bioactive metabolites with potent antimicrobial and insecticidal properties. Recent studies have shown that *Bv* has significant larvicidal activity against a variety of mosquito species, making it a strong candidate for mosquito management [9,10]. The bacterium produces a complex array of secondary metabolites, including lipopeptides, polyketides, and hydrolases, which effectively disrupt larval development and significantly reduce mosquito population density [11]. In addition, *Bv* is characterized by its environmental friendliness, as its highly selective mode of action minimizes unintended ecological consequences and it has low toxicity to non-target organisms, making it a sustainable and safe alternative for mosquito control [12]. Due to its high efficacy and ecological safety, *Bv* represents a transformative tool for integrated vector management strategies, with the potential to improve current mosquito control efforts and limit the spread of mosquito-borne diseases.

Despite the promising mosquitocidal potential of *Bv*, its effects on the mosquito life history traits remain poorly understood. While it is known that *Bv* can reduce mosquito populations by directly killing larvae, less attention has been paid to potential sublethal effects on surviving individuals. Sublethal exposures may result in significant but less obvious consequences, such as altered developmental duration, reduced adult emergence, and impaired reproductive success, all of which can affect mosquito population dynamics and vectorial capacity [13,14]. Understanding these sublethal effects is critical, as they could complement larvicidal efficacy by further suppressing mosquito populations and altering disease transmission potential. Therefore, this study aimed to investigate the lethal and sublethal effects of *Bv* strain WHk23 isolated from Wadi Hanifah against *Cx. quinquefasciatus*, the main vector of arboviral and parasitic diseases. Specifically, mosquito mortality at different concentrations was evaluated to assess larvicidal efficacy while examining key life history traits, including development time, adult emergence, survival, and reproductive capacity, to fully understand its potential as a biocontrol agent.

## 2. Materials and Methods

### 2.1. Mosquito Rearing

The *Cx. quinquefasciatus* colony used in this experiment was reared and maintained under standard insectary conditions at 27 ± 1 °C, 60 ± 10% relative humidity, and a 12:12 h light–dark photoperiod. Eggs were hatched in plastic trays containing 1.5 L of dechlorinated water. Larvae were reared at a density of 300 individuals per tray and fed 0.2 g (approximately 0.67 mg/larva) of finely ground TetraMin fish food (Tetra Holding Inc., Blacksburg, VA, USA) every two days, except during the 24 h exposure period to the test toxin. Each pupa was individually transferred to a 30 mL plastic cup with dechlorinated water and maintained until adult emergence. Newly emerged adults were transferred to insect rearing cages (30 cm^3^, BioQuip Products, Rancho Dominguez, CA, USA) and provided with 10% sucrose solution via dental cotton rolls. Female mosquitoes were blood-fed once using defibrinated chicken blood delivered through the Hemotek membrane feeding system (Hemotek Ltd., Blackburn, UK) for egg production.

### 2.2. Preparation of Bacterial Crude Toxins

The strain of *Bv* WHk23 was isolated from a soil sample collected in Wadi Hanifah, Riyadh, as described previously [9]. The bacteria were inoculated into 300 mL of NB2 in a sterile 1 L flask and incubated at 30 °C for two nights with shaking at 200 rpm. The culture was then centrifuged at 13,000× *g* for 10 min at 4 °C to obtain the supernatant. The resulting supernatant was acidified to pH 2 with 6N HCl and stored at 4 °C overnight to facilitate the precipitation of the crude toxin. The precipitate was collected by centrifugation at 9000× *g* at 4 °C for 30 min. The resulting precipitate was resuspended in dH_2_O, and the pH was adjusted to 7 with 1N NaOH to ensure solubilization. The solubilized original toxin was then lyophilized, weighed, and stored at 4 °C until being used for experiments.

### 2.3. Toxicity Bioassay

A 1 mg/mL stock solution of *Bv* crude toxin was prepared with dechlorinated water and then serially diluted to test the susceptibility of larvae of *Cx. quinquefasciatus* to *Bv*. Several concentrations of the crude toxin were tested (5, 10, 20, 35, 50, 65, 70, 90, and 100 µg/mL). Twenty third instar larvae were placed in plastic cups containing 200 mL of dechlorinated water with the desired toxin concentration. Each concentration was replicated three times and incubated at a controlled temperature of 27 °C. Larval mortality was recorded after 24 h of exposure. If the mortality of the control group exceeded 20%, the results were considered invalid, and the experiment was repeated. Based on the concentration–response data from the bioassay, the lethal concentrations (LC_30_, LC_50_, and LC_70_) for subsequent experiments were determined using probit analysis.

### 2.4. Larval Development Time and Adult Emergence

**Figure 1:**
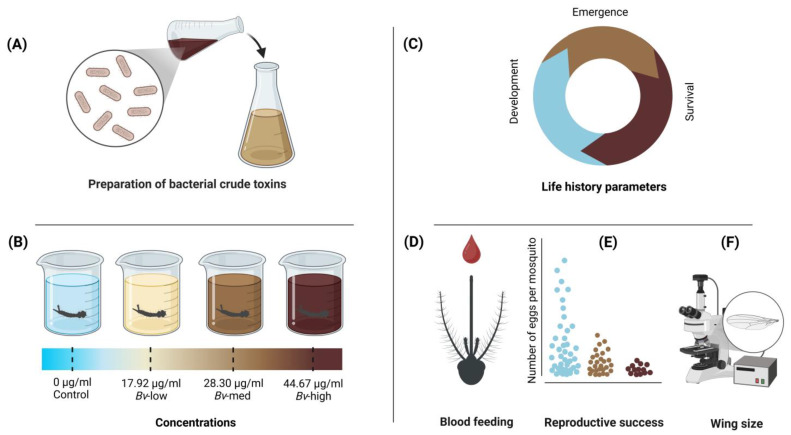
Schematic overview of experimental workflow. Crude toxin preparation of *Bv* for mosquito concentration–response studies (**A**). Following the estimation of lethal concentrations, mosquito larvae were subject to four levels of treatments (control, *Bv*-low (LC_30_), *Bv*-medium (LC_50_), and *Bv*-high (LC_70_) (**B**). Life history parameters (e.g., development time, adult emergence, female survival rates) were determined for mosquitoes from each experimental treatment (**C**). Adult female mosquitoes from each of the four treatments were orally bloodfed for egg production (**D**). Reproductive success (fecundity and fertility) was determined (**E**). Adult female body sizes were estimated by measuring wing length (**F**).

Three hundred first instar larvae (<24 h old) were placed into each experimental tray (experimental unit) containing 1.5 L of dechlorinated water with 0.2 g of larval food (TetrMin fish food). The experimental trays were assigned to one of four treatments—control, *Bv*-low (LC_30_), *Bv*-medium (LC_50_), and *Bv*-high (LC_70_) concentrations of *Bv*—and replicated three times (Figure 1).

These concentrations were applied to *Bv* treatments when the majority reached the third instar stage, while control groups received no *Bv* exposure. During the 24 h exposure period, no food was provided for the larvae. After this period, surviving larvae from each treatment were transferred to new trays containing dechlorinated water without toxins. New pupae were collected daily and placed individually in 30 mL plastic cups for adult emergence. After emerging, adults were fed a 10% sucrose solution. For each experimental tray, larval development time and adult emergence (total number of adults emerged divided by the initial number of larvae introduced, expressed as a percentage) were monitored daily. All experimental trays were kept under controlled conditions with a 12 h light–dark photoperiod, a temperature of 27 °C, and a relative humidity of 60 ± 10%.

### 2.5. Female Survival, Reproductive Fitness, and Offspring Viability

Female survival was assessed by placing newly emerged female mosquitoes into 16 oz paper-cup cages. Access to 10% sucrose solution was provided by a cotton ball placed on the top of the cages and changed every three days. Female survival was monitored daily. The female cages were kept at 27 °C until the end of the survival experiment. To assess the effect of *Bv* on mosquito reproduction (fecundity), emerged adults from each treatment group were first maintained in mesh cages with both males and females together to allow for natural mating. Subsequently, a group of five- to eight-day-old female mosquitoes were deprived of sucrose overnight, and then offered a single blood meal consisting of defibrinated chicken blood for 1 h using a Hemotek membrane blood feeder preheated to 37 °C. After feeding, mosquitoes were immobilized by cold treatment (2–3 min), and partially fed and unfed individuals were discarded. Fully engorged females from each treatment group were placed individually in new cup cages with mesh screens and provided with 10% sucrose solution via cotton balls. Each cage contained a 100 mL plastic oviposition cup filled with dechlorinated water. The total number of eggs laid by each female mosquito was recorded as a measure of fecundity. Fertility was assessed approximately 72 h after oviposition by counting the number of neonates hatched from each egg raft.

### 2.6. Female Body-Size Determination

To assess the effects of *Bv* on mosquito body size, groups of female mosquitoes were collected from each treatment and body size was determined by measuring wing length as a proxy for body size [15]. One wing from each female was selected, mounted on a microscope slide, and the distance from the axillary margin to the apical notch without considering wing fringe was measured in millimeters. Measurements were made using a calibrated dissecting microscope equipped with an ocular micrometer.

### 2.7. Data Analysis

Concentration–response data were analyzed using probit analysis, corrected for control mortality, when necessary, to estimate LC_30_, LC_50_, and LC_70_ values and their 95% confidence intervals. Data on the effects of exposure on mosquito life history, including development time, adult emergence, fecundity, fertility, and wing length, were analyzed using separate ANOVA tests followed by multiple comparisons using Tukey post hoc tests. Adult survival data of females were analyzed using a Cox proportional hazards regression analysis of survival data. Statistical analyses were performed using GraphPad Prism 10 (GraphPad Software, San Diego, CA, USA), with a *p* value of <0.05 considered statistically significant.

## 3. Results

### 3.1. Toxicity Bioassay, Larval Development Time, Adult Emergence, and Female Body Size

**Figure 2:**
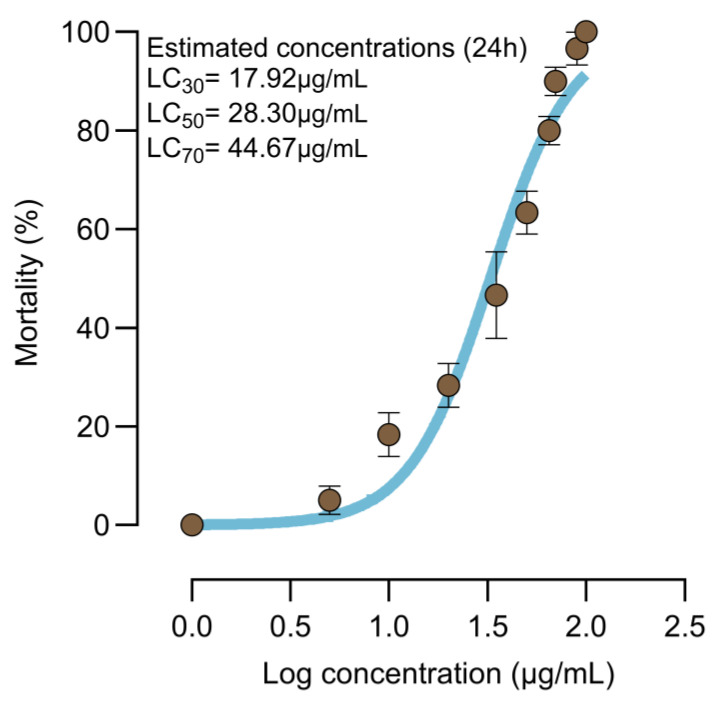
Concentration–response curve of *Bv* for larval mortality. Concentrations represented as log_10_ transformation of µg/mL. The data shown represent means and the bars indicate standard error of the means. The light blue solid line represents the best fit to data. Each brown circle represents an average larval mortality data from a single concentration–response experiment performed in triplicate. Estimated lethal concentrations are displayed on the plot.

Bioassays were performed on third instar *Cx. quinquefasciatus* larvae exposed to different concentrations of *Bv* for 24 h. Concentration–response analysis confirmed the lethal effect of *Bv* with estimated lethal concentration (LC) values and their 95% confidence intervals as follows: LC_30_ = 17.92 µg/mL (11.67–23.68), LC_50_ = 28.30 µg/mL (21.00–36.14), and LC_70_ = 44.67 µg/mL (34.97–59.53) (Figure 2). These concentrations were used to assess sublethal effects on mosquito life history traits after 24 h of exposure.

**Figure 3:**
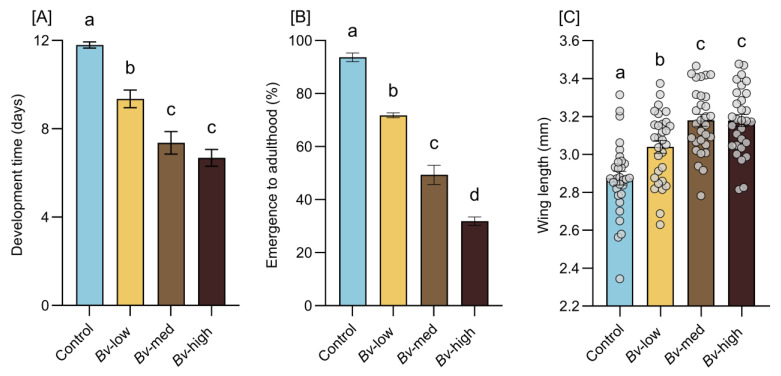
Treatment effects on larval development time (**A**), emergence to adulthood (**B**), female wing length (**C**). Bars represent means  ±  standard error of the means. In panel (**C**), each dot represents data from an individual mosquito. Different letters indicate statistically significant differences between treatment groups (*p* < 0.05).

Larval development time significantly differed between treatments (F = 36.20, *p* < 0.0001). The longest duration was observed in the control group, while development time was significantly shorter in larvae exposed to *Bv* treatments (*Bv*-low, *Bv*-medium, and *Bv*-high) (Figure 3A).

There was a significant difference in development time between the control and exposed groups, but no significant difference was found between the *Bv*-medium and *Bv*-high groups (*p* > 0.05). The adult emergence rates were highest in the control group, while exposure to *Bv* resulted in a significant, dose-dependent decrease in the emergence rates (Figure 3B). There was a significant difference in emergence rates between the control and exposed groups (F = 152.6, *p* < 0.0001). Wing length measurements showed that exposure to *Bv* during the larval stage resulted in a significant increase in adult body size in comparison to controls (F = 19.01, *p* < 0.0001) (Figure 3C). However, no statistically significant differences in wing length were found between females in the *Bv*-medium and *Bv*-high groups (*p* > 0.05).

### 3.2. Female Survival, Reproductive Fitness, and Offspring Viability

**Figure 4:**
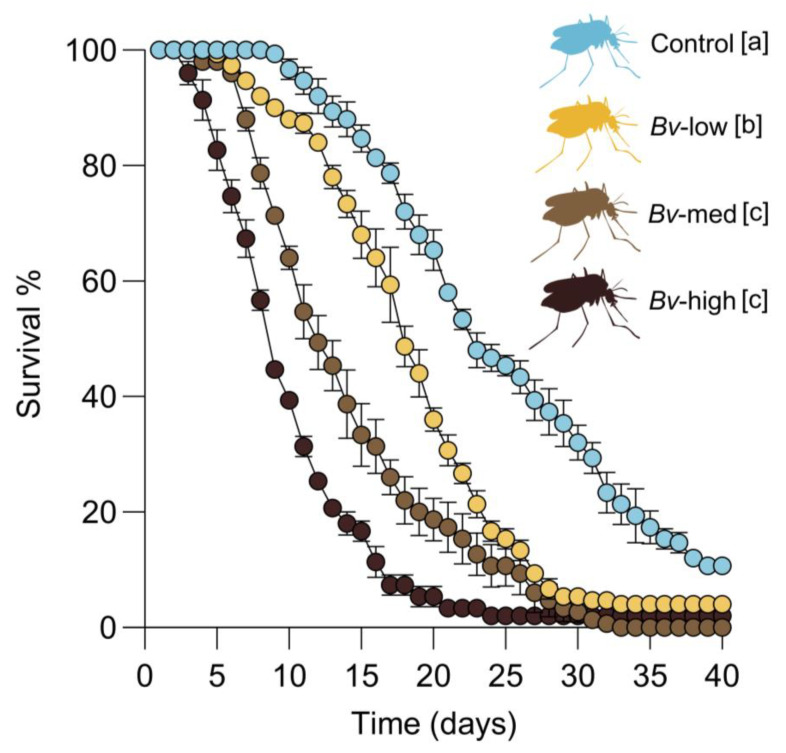
Survival curves of female mosquitoes following the exposure to *Bv* at the larval stage. Data shown represent the means ± standard error of the means. Different letters next to treatment names indicate statistically significant differences between treatment groups (*p* < 0.05).

Regression analysis revealed a significant effect of *Bv* treatment on female survival (χ^2^ = 146.0, *p* < 0.0001). Exposure of third instar larvae to *Bv*-low, *Bv*-medium, and *Bv*-high concentrations significantly reduced the lifespan of female mosquitoes in comparison to controls, with greater reductions observed at higher concentrations. Larvae treated with *Bv*-high concentrations had the lowest adult survival compared to those treated with *Bv*-low or *Bv*-medium concentrations. Survival analysis showed that the survival curves were statistically significant between *Bv*-low vs. *Bv*-medium and *Bv*-low vs. *Bv*-high concentrations, whereas the curves were not statistically significant between *Bv*-medium and *Bv*-high concentrations (*p* > 0.05) (Figure 4).

**Figure 5:**
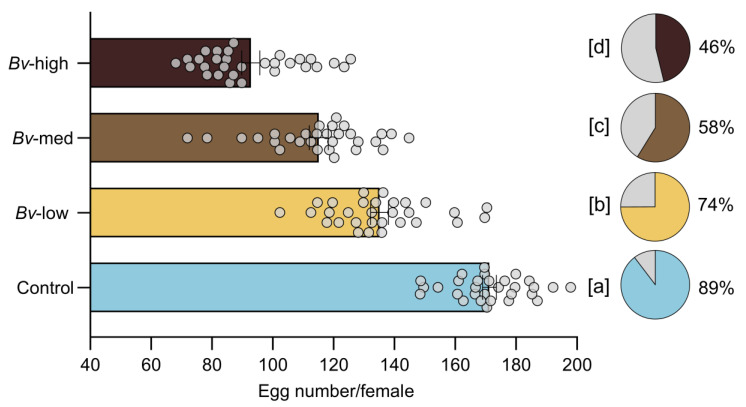
Fecundity and fertility for each treatment group. Bars represent means  ±  standard error of the means. Each dot represents a single mosquito sample. Filled areas on pie charts and numbers represent the percentage of hatchability (fertility). Different letters indicate statistically significant differences between treatment groups (*p* < 0.05).

ANOVA showed a significant effect of *Bv* on female fecundity (F = 136.9, *p* > 0.0001) and fertility (F = 490.5, *p* < 0.0001) following exposure to *Bv* at the larval stage. A concentration-dependent decrease in reproductive fitness was observed, with females exposed to high concentrations of *Bv* laying significantly fewer eggs (Figure 5).

Egg viability followed the same pattern, with a higher hatching rate in the control group compared to the exposed groups (Figure 5). Fecundity and fertility were significantly reduced in the exposed groups compared to the counterparts in control, with statistical differences observed between treatment groups.

## 4. Discussion

Biological control agents represent a promising alternative to conventional chemical insecticides for mosquito control. *Bacillus velezensis* has emerged as a potential biocontrol agent due to its ability to produce a wide range of secondary metabolites and bioactive compounds with insecticidal properties. Although it is not as extensively studied or widely used as *Bti*, recent research indicates that *Bv* may hold significant promise in targeting mosquito larvae and reducing vector populations [9,10].

The third instar larval toxicity bioassay revealed that the LC_50_ of *Bv* against *Cx. quinquefasciatus* was estimated to be 28.30 µg/mL after 24 h of exposure, supporting previous findings on the biocontrol potential of this strain. Consistent with this result, the same strain has been reported to exhibit significant larvicidal activity, with LC_50_ values of 25.41 and 33.97 µg/mL against *Anopheles stephensi* and *Cx. quinquefasciatus*, respectively [9]. A study by Kannan Revathi et al. [16] demonstrated that the culture supernatant of *B. subtilis* exhibited significant larvicidal activity against *Aedes aegypti,* with LC_50_ values of 1.73 µg/mL. Similarly, *B. amyloliquefaciens* culture supernatant showed species-specific toxicity, with LC_50_ values of 26.4 µg/mL for *An. stephensi*, 22.2 µg/mL for *Cx. quinquefasciatus*, and 20.5 µg/mL for *Ae. aegypti* [17]. In comparison, *Bv* strains B64a and B15 exhibited larvicidal activity against *Ae. aegypti*, with crude lipopeptide extracts reaching LC_50_ values of 110 µg/mL and 120 µg/mL, respectively, where the authors suggested that the larvicidal effect was mainly due to metabolites produced by these bacteria [12]. Furthermore, silver nanoparticles synthesized using *Bv* demonstrated strong toxicity against third instar *Ae. aegypti* larvae, with an LC_50_ of 0.76 µg/mL [10]. These findings highlight the potential of *Bv* as an effective larvicidal agent, although its efficacy varies depending on the strain, mosquito species, and extraction or formulation method. This underscores the need for careful strain selection and optimization to enhance biocontrol efficacy against mosquito vectors. 

Several studies have shown that sublethal exposure to *Bacillus* spp. and chemical insecticides during larval stages can result in a carry-over effect on the survival and fitness of adult mosquitoes. For instance, *Ae. aegypti* larvae exposed to *Bti* developed into adults with reduced survival rates [18]. Similarly, a study examined the effects of sublethal permethrin exposure on various fitness parameters of *Cx. quinquefasciatus*, where the exposure resulted in a significant reduction in the survival of adult females. These detrimental effects are suggested to result from physiological stress and energy costs associated with detoxification processes, ultimately compromising the overall fitness and survival of adult mosquitoes [19]. In another study, larval exposure to pyriproxyfen resulted in reduced survival of *Ae. aegypti* adults [20]. In the current study, the sublethal exposure to *Bv* significantly shortened the survival of *Cx quinquefasciatus* females, with the effect being more pronounced at higher exposure concentrations. This indicates a potential dose-dependent physiological cost associated with larval exposure to *Bv*, which may impact female survival. Further studies are needed to elucidate the exact mechanism responsible for the reduction in female survival following the larval exposure. Collectively, these findings underscore the importance of considering sublethal effects in mosquito control programs. Beyond direct larvicidal activity, biocontrol agents such as *Bv* may also contribute to population suppression through a reduction in adult mosquito survival, which could have implications for vector control efficacy and disease transmission dynamics.

The current study demonstrated that exposure to *Bv* led to a dose-dependent larval mortality, reducing intraspecific competition for food and space. This reduction in larval competition contributed to a shortened development time and the emergence of larger adults [21]. These effects are consistent with previous findings where exposure to chemical agents such as malathion resulted in larval mortality that promoted faster development and larger adult body size [22,23]. Under high densities, competition prolongs development and often results in smaller adults, whereas reduced competition promotes faster development and yields larger individuals with the potential for increased reproductive output. However, no fitness advantage was observed in terms of reproductive success in the emergence of larger females following *Bv* exposure. Contrary to the common assumption that larger mosquitoes exhibit higher fecundity, this study revealed that exposure to *Bv* negatively affected both fecundity and fertility, leading to fewer eggs laid and lower hatching rates. These results align with earlier observations in *An. coluzzii*, where larval exposure to increasing concentrations of *Bti* produced larger adults but was accompanied by a decline in egg production [24]. Similar trends have been reported in other studies, where larval exposure to *Bti* resulted in reductions in fecundity of *Ae. albopictus* [18]. Although this study did not directly investigate the underlying mechanisms of sublethal effects, these findings suggest that larval exposure to *Bv* may influence not only mosquito development and size but also induce physiological effects that impair reproductive capacity. These results underscore the complex trade-offs associated with sublethal exposure to insecticides, where increased body size does not necessarily translate into enhanced fecundity [15,25]. Understanding such effects is critical for evaluating the broader implications of *Bv* as a biocontrol agent and for optimizing its integration into mosquito management programs.

## 5. Conclusions

This study demonstrates that exposure to *Bv* caused both direct and indirect effects on mosquito populations, highlighting its potential as a novel biocontrol agent for mosquito vector management. Directly, this bacterium induces larval mortality, thereby reducing overall population size. Indirectly, sublethal exposure during the larval stage compromises key adult life history traits, including female survival and reproductive capacity. These effects collectively can reduce the vectorial capacity of mosquito populations by limiting the number of long-lived, reproductively viable females that are primarily responsible for disease transmission. Given these promising outcomes, further research focused on optimizing *Bv* formulations and integrating them into existing vector control management programs may enhance the efficacy of mosquito borne-disease prevention strategies.

## Data Availability

The original contributions presented in this study are included in the article. Further inquiries can be directed to the corresponding author.
